# Knockdown of m6A Reader IGF2BP3 Inhibited Hypoxia-Induced Cell Migration and Angiogenesis by Regulating Hypoxia Inducible Factor-1α in Stomach Cancer

**DOI:** 10.3389/fonc.2021.711207

**Published:** 2021-09-21

**Authors:** Libin Jiang, Yingxia Li, Yixin He, Dapeng Wei, Lvyin Yan, Hongtao Wen

**Affiliations:** Department of Gastroenterology, The First Affiliated Hospital of Zhengzhou University, Zhengzhou, China

**Keywords:** stomach cancer, hypoxia, migration, angiogenesis, IGF2BP3, HIF-1A

## Abstract

Hypoxia is a common feature of solid tumors including stomach cancer (SC) and is closely associated with cancer malignant progression. N6-methyladenosine (m6A), a common modification on RNA, is involved in the regulation of RNA fate and hypoxic responses in cancers. However, the interaction between m6A reader insulin-like growth factor-II mRNA-binding protein 3 (IGF2BP3) and SC hypoxic microenvironment is poorly defined. In the present study, expression levels of IGF2BP3 and hypoxia inducible factor-1α (HIF1A) were examined by bioinformatics analysis and RT-qPCR and western blot assays. Cell migratory ability was assessed through Transwell and wound healing assays. The angiogenic potential was evaluated by VEGF secretion, tube formation, and chick embryo chorioallantoic membrane (CAM) assays. The interaction between IGF2BP3 and HIF1A was explored using bioinformatics analysis and RIP and luciferase reporter assays. The results showed that IGF2BP3 and HIF1A were highly expressed in SC tissues and hypoxia-treated SC cells. IGF2BP3 knockdown inhibited hypoxia-induced cell migration and angiogenesis in SC. IGF2BP3 positively regulated HIF1A expression by directly binding to a specific m6A site in the coding region of HIF1A mRNA in SC cells. HIF1A overexpression abrogated the effects of IGF2BP3 knockdown on hypoxia-induced cell migration and angiogenesis in SC. In conclusion, IGF2BP3 knockdown inhibited hypoxia-induced cell migration and angiogenesis by down-regulating HIF1A in SC.

## Introduction

Stomach cancer (SC) is the fifth commonest cancer and the third most deadly cancer globally (1, 2). It was estimated that SC was responsible for about 6% of all new cancer diagnoses and approximately 8% of all cancer-related deaths in 2018 worldwide (1, 2). Stomach adenocarcinoma (STAD), the most common SC type, is highly treatable when it is diagnosed at early stages (3, 4). However, many SC cases are diagnosed at advanced stages and the prognosis of SC patients with advanced disease is poor with a median survival of approximately 1 year (3–5). It is well known to us that the high mortality of cancers can be largely attributed to the metastasis of tumor cells, which involves multiple biological processes such as cell growth, migration, invasion, and angiogenesis (6). The hypoxic microenvironment, a universal hallmark of almost all malignancies including SC, is closely linked with tumor metastasis (7). In the hypoxic environment, hypoxic tumor cells undergo multiple alterations at the molecular, cellular, and phenotypic levels (*e.g.* increased migration, invasion, and angiogenesis) that can help them to survive in primary and secondary sites or escape from the unfavorable tumor environment (8, 9). Moreover, hypoxia can activate a series of adaptive responses that can facilitate the tumorigenesis and progression of cancers (10). Additionally, hypoxia has been reported to be a mediator of intratumor heterogeneity and a driver of tumor progression, immune escape, and therapeutic resistance (11–13). Thus, it is imperative to investigate the pathogenesis of SC in a hypoxic microenvironment.

N6-methyladenosine (m6A), the most common modification on eukaryotic RNA, can regulate RNA expression by influencing almost all aspects of the RNA life cycle such as splicing, processing, stability, and translation (14, 15). M6A modification level can be altered by m6A methyltransferases (“writers”) and demethylases (“erasers”) and recognized by some m6A binding proteins (“readers”) (14, 16). Recently, accumulating evidence shows that these m6A regulators play central roles in the growth and aggressive progression of tumors (16–18). Moreover, some studies have demonstrated that hypoxia can trigger alterations in the levels or activities of m6A regulators (19, 20). For instance, Panneerdoss et al. pointed out that ALKBH5 (m6A “eraser”) and METTL14 (m6A “writer”) expression levels were markedly increased and YTHDF3 (m6A “reader”) level was notably reduced in hypoxia-treated breast cancer cells compared with the normoxia group (19).

Insulin-like growth factor-II mRNA-binding protein 3 (IGF2BP3), also named IMP3, is an m6A “reader” belonging to the IGF2 mRNA-binding protein family (21, 22). IGF2BP3 has been found to be highly expressed and to be a potential oncogenic factor in plenty of cancers including SC (23–25). Moreover, SC patients with a high IGF2BP3 expression had a poor prognosis (26–28). Additionally, previous studies suggested that IGF2BP3 might be involved in the regulation of hypoxic responses (29, 30). For instance, Huang et al. showed that IGF2BP3 could regulate the alternative splicing of multiple hypoxia-responsive genes (30).

In this text, the roles of IGF2BP3 in hypoxia-induced cell migration and angiogenesis together with its m6A-dependent targets were investigated in SC *in vitro* and *in vivo*.

## Materials and Methods

### Clinical Samples

A total of 20 pairs of SC tissues and adjacent normal tissues were obtained from the First Affiliated Hospital of Zhengzhou University. Our study was approved by the Research Ethics Committee of the First Affiliated Hospital of Zhengzhou University. Also, we got the written informed consents from all patients.

### Reagents

Small interference RNAs (siRNAs) were ordered from GenePharma Co., Ltd. (Shanghai, China). IGF2BP3 or HIF1A coding region was respectively sub-cloned into pcDNA3.1 empty vector by Wuhan GeneCreate Biological Engineering Co., Ltd. (Wuhan, China) to generate pcDNA-IGF2BP3 or pcDNA-HIF1A overexpression plasmid. The sequences of siRNAs targeting IGF2BP3 were shown as below: 5’-CCUUGAAAGUAGCCUAUAUTT-3’ for si-IGF2BP3#1; 5’-GCAGGAAUUGACGCUGUAUTT-3’ for si-IGF2BP3#2; 5’-GCUGGAGCUUCAAUUAA GATT-3’ for si-IGF2BP3#3.

### Cell Culture and Transfection

MKN-45 cells and human umbilical vein endothelial cells (HUVECs) were purchased from the China Center for Type Culture Collection (Wuhan, China). HGC-27 cells were obtained from the Cell Bank of the Chinese Academy of Sciences (Shanghai, China). MKN-45 and HGC-27 cells were maintained in RPMI-1640 medium (Thermo Scientific, Waltham, MA, USA) containing 10% fetal bovine serum (Thermo Scientific). HUVECs were maintained in complete endothelial cell medium (ECM) (Sciencell Research Laboratories, Carlsbad, CA, USA). Hypoxic conditions: 1% O_2_, 94% N_2_, 5% CO_2_, and 37°C. Normoxic conditions: 95% air, 5% CO_2_, and 37°C. siRNAs or overexpression plasmids were transfected into MKN-45 and HGC-27 cells using Lipofectamine 3000 Transfection Reagent (Thermo Scientific).

### Total RTA Isolation and m6A Methylation Level Determination

Total RNA was isolated from SC tissues, adjacent normal tissues, and SC cells using Trizol reagent (Thermo Scientific) following the manufacturer’s protocols. Next, the cDNA first strand was synthesized using M-MLV Reverse Transcriptase (Thermo Scientific) and then amplified and quantified using SYBR Green PCR Master Mix (Thermo Scientific) together with specific quantitative primers. The primer sequences were shown as follows: 5’-GTCAAGTGCAGAAGTTGTTGTC-3’ (forward) and 5’-GCAATCTGTCTTTGGTTTGGC-3’ (reverse) for IGF2BP3; 5’-TCCAAGAAGCCCTAACGTGT-3’ (forward) and 5’-TGATCGTCTGGCTGCTGTAA-3’ (reverse) for HIF1A; 5’-ATTGCCGACAGGATGCAGA-3’ (forward) and 5’-GAGTACTTGCGCTCAGGAGGA-3’ (reverse) for β-actin. β-actin acted as the endogenous reference to normalize the expression of IGF2BP3 and HIF1A. The relative expression levels of IGF2BP3 and HIF1A were calculated using the 2^-ΔΔCt^ method. Global RNA m6A methylation level was determined using an EpiQuik M6A RNA Methylation Quantification Kit (Farmingdale, NY, USA) according to the instructions of the manufacturer.

### Western Blot Assay

The lysates of MKN-45 and HGC-27 cells were prepared using RIPA lysis buffer (Beyotime Biotechnology Co., Ltd., Shanghai, China) containing the protease inhibitor (Thermo Scientific). Protein contents were measured using the Pierce BCA Protein Assay Kit (Thermo Scientific). An equal amount of proteins (30μg per lane) was separated through SDS-PAGE and then transferred onto PVDF membranes (Millipore, Bedford, MA, USA). After blocked with 5% non-fat milk, the membranes were incubated overnight at 4˚C with anti-IGF2BP3 (1/2000 dilution, ab177477, Abcam, Cambridge, UK), anti-HIF1A (1/1000 dilution, ab179483, Abcam), or anti-β-actin (1/2000 dilution, ab8227, Abcam). After washed, the membranes were incubated for 1 h at room temperature with a secondary antibody conjugated with horseradish peroxidase (1/5000 dilution, ab6721, Abcam). Finally, protein bands were developed using the Pierce ECL Western Blotting Substrate (Thermo Scientific).

### Transwell Migration Assay

The upper or low chambers of transwell plates (8µm pore-size filter membranes; Corning Inc., New York, NY, USA) were added with cells suspended in serum-free medium or medium containing 20% FBS, respectively. Next, the transwell plates were placed in normoxic or hypoxic conditions. Cells migrated onto the bottom side of the membranes were fixed with 4% paraformaldehyde, stained with 1% crystal violet (Sigma-Aldrich, St Louis, MO, USA), and imaged using a microscope. Finally, the number of migrated cells was calculated in 5 random fields.

### Wound Healing Assay

Cells were transfected with or without siRNAs/plasmids and cultured to 100% confluency under normoxic conditions. The cell monolayer was scratched using a pipette tip to generate a vertical scratch wound. Next, cells were exposed to hypoxia or normoxia for 24 h. The marked wound areas were imaged using a microscope. The horizontal wound widths were determined in 5 random points of each wound.

### Vascular Endothelial Growth Factor (VEGF) Level Determination

VEGF level in the culture supernatants of MKN-45 and HGC-27 cells was measured using the human VEGFA ELISA kit (Abcam) following the manufacturer’s protocols.

### Tube Formation Assay

MKN-45 and HGC-27 cells transfected with or without si-NC or si-IGF2BP3 were exposed to normoxia or hypoxia, followed by the collection of cell culture supernatants (conditioned medium). HUVECs were plated in Matrigel (Corning)-coated 96-well plates and cultured in the mixed medium of concentrated conditioned medium derived from SC cells and ECM medium (volume ratio=1:1). Next, the tube formation capacity of HUVECs was evaluated at 12 h after incubation.

### RNA Immunoprecipitation (RIP) Assay

RIP assay was carried out in MKN-45 cells using the Magna RIP RNA-Binding Protein Immunoprecipitation Kit (Millipore, Temecula, CA, USA) and antibody against IgG or IGF2BP3 according to the manufacturer’s protocols. HIF1A level enriched by IgG or IGF2BP3 antibody was examined by RT-qPCR assay.

### Luciferase Reporter Assay

The partial coding region (CDS) of HIF1A covering putative IGF2BP3 binding site was constructed into a pGL3-Basic plasmid by Wuhan GeneCreate Biological Engineering Co., Ltd. (Wuhan, China) to generate WT HIF1A recombinant reporter. Also, MUT HIF1A recombinant reporter containing mutant IGF2BP3 binding site was produced by Wuhan GeneCreate Biological Engineering Co., Ltd. MKN-45 cells were co-transfected with the recombinant reporter, pRL-TK Renilla luciferase plasmid, and pcDNA3.1/pcDNA-IGF2BP3. At 48 h after co-transfection, Dual-Luciferase Reporter Assay (Promega, Madison, WI, USA) kit was used to detect the luciferase activities with Renilla luciferase activity as the internal control.

### Chick Embryo Chorioallantoic Membrane (CAM) Assay

Fertilized chicken eggs were incubated for 7 days at 37°C in a 60% humidity incubator. Next, a small hole was made in the air sac of eggs and a small window was produced in the shell of eggs. Next, physiological saline was inoculated and the ventricular membrane was separated from the CAM. After the removal of the ventricular membrane, a rubber ring was placed onto the exposed CAM. Next, the concentrated culture medium isolated from treated/transfected SC cells was added into a rubber ring. The windows were sealed using the sealing film. After 3 days of incubation at 37°C, the area around the rubber ring was imaged and the pattern of angiogenesis was estimated.

### Statistical Analysis

Data analysis was conducted using SPSS 20.0 software (SPSS, Chicago, IL, USA). The outcomes were presented as mean ± standard deviation. The student’s *t*-test was used to analyze the difference between groups. One-way analysis of variance (ANOVA) along with Tukey’s *post-hoc* test was utilized to examine the difference among groups. The difference was regarded as statistically significant at *P <*0.05.

## Results

### M6A Regulator IGF2BP3 Was Highly Expressed in SC Tissues

**Figure 1:**
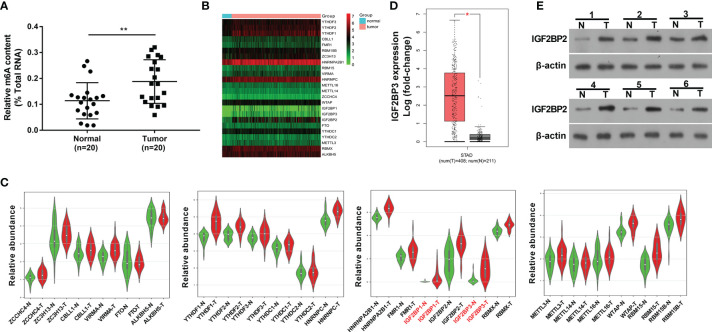
M6A regulator IGF2BP3 was highly expressed in SC tissues. **(A)** Global m6A methylation level in total RNA was measured in 20 pairs of SC tumor tissues and adjacent normal tissues. **(B)** Expression heatmap of 24 m6A-related genes in 303 cases of SC tumor tissues and 32 cases of normal tissues. **(C)** Violin plots of 24 m6A-related genes in 27 pairs of SC tumor tissues and adjacent normal tissues. Violin plots were drawn using the bioinformatics website (http://www.bioinformatics.com.cn/plot_basic_vertical_horizontal_violin_plot_068). **(D)** Expression analysis of IGF2BP3 in SC tumor tissues (n=408) and normal tissues (n=211). The figure was drawn by the GEPIA website (http://gepia.cancer-pku.cn/). **(E)** Expression level of IGF2BP3 in 6 pairs of SC tumor tissues and adjacent normal tissues was measured by western blot assay. ^*^ *p* < 0.05, ^**^ *p* < 0.01.

Recently, RNA m6A methylation modification and related regulators have attracted much attention from researchers given their crucial roles in plenty of cancers (14). In this text, we demonstrated that the global m6A level in total RNA was markedly increased in SC tumor tissues (n=20) than that in adjacent normal tissues (n=20) (**Figure 1A**), suggesting that the alteration of RNA m6A modification level was closely linked with the tumorigenesis and progression of SC. To identify vital genes implicated in the pathogenesis of SC, gene expression profiles in SC tumor tissues and normal tissues were downloaded from the UCSC Xena website (http://xena.ucsc.edu/). The expression heatmap of 24 m6A-related genes in SC tumor tissues and normal tissues was presented in **Figure 1B**. Differential expression analysis for these 24 m6A-related genes revealed that only IGF2BP1 and IGF2BP3 were highly expressed (|log_2_FoldChange| > 1, *P*<0.05) in SC tumor tissues relative to the normal group (**Figure 1C**). Moreover, the GEPIA website (http://gepia.cancer-pku.cn/) analysis showed that there was a noticeable increase of IGF2BP3 expression level in 408 cases of SC tumor tissues than that in 211 cases of normal tissues (|log_2_FoldChange| > 1, **Figure 1D**). Moreover, western blot assay showed that IGF2BP3 protein expression level was notably increased in 6 SC tumor tissues compared to matched adjacent normal tissues (**Figure 1E**). Thus, IGF2BP3 was selected for further investigation.

### IGF2BP3 Knockdown Inhibited Hypoxia-Induced Cell Migration and Angiogenesis in SC

**Figure 2:**
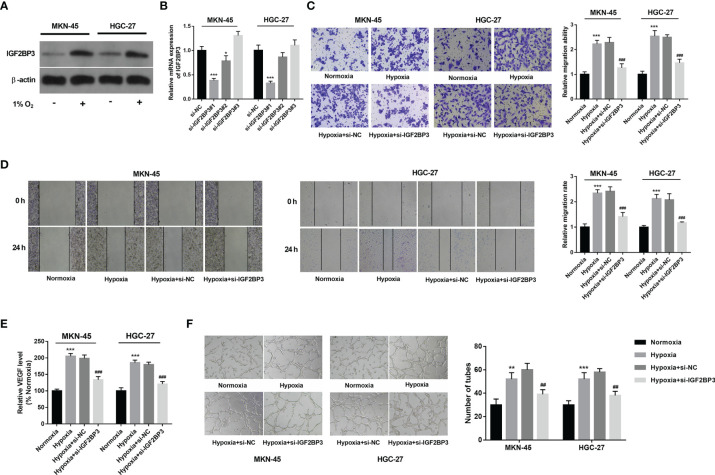
IGF2BP3 knockdown hampered hypoxia-induced cell migration and angiogenesis in SC. **(A)** MKN-45 and HGC-27 cells were cultured in normoxic or hypoxic conditions for 24 h Next, the IGF2BP3 protein level was measured by western blot assay. **(B)** MKN-45 and HGC-27 cells were transfected with si-NC, si-IGF2BP3#1, si-IGF2BP3#2, or si-IGF2BP3#3. Next, the IGF2BP3 mRNA level was measured by RT-qPCR assay at 48 h after transfection. **(C–F)** MKN-45 and HGC-27 cells were transfected with si-NC or si-IGF2BP3#1 for 48 h and then maintained in hypoxic conditions for another 24 h Cells in the normoxia group were maintained in normoxic conditions for 72 h Cells in the hypoxia group were cultured in normoxia for 48 h and then exposed to hypoxia for an additional 24 h **(C, D)** Cell migratory potential was assessed by Transwell migration **(C)** and wound healing **(D)** assays. **(E)** VEGF level in cell culture supernatants was detected using a commercial kit. **(F)** The conditioned medium of MKN-45 and HGC-27 cells were collected after normoxia/hypoxia treatment or/and transfection. Next, HUVECs were cultured in a mixed medium of ECM and conditioned medium (volume ratio=1:1), followed by the measurement of tube formation ability at 12 h after incubation. * indicate that the difference is significant at 0.05 level. ^**^ *p* < 0.01, ^***^ *p* < 0.001, ^##^ *p* < 0.01, ^###^ *p* < 0.001 compared with the normoxia group.

Western blot assay demonstrated that IGF2BP3 expression was notably increased in hypoxia-treated MKN-45 and HGC-27 cells than that in cells exposed to normoxia (**Figure 2A**). To further explore the roles of IGF2BP3 in SC progression under hypoxic conditions, 3 siRNAs targeting IGF2BP3 (si-IGF2BP3#1, si-IGF2BP3#2, and si-IGF2BP3#3) and a control siRNA (si-NC) were synthesized. Knockdown efficiency analysis revealed that the transfection of si-IGF2BP3#1 or si-IGF2BP3#2 led to the notable reduction of IGF2BP3 expression level in MKN-45 and HGC-27 cells compared to the si-NC group (**Figure 2B**). Next, si-IGF2BP3#1 was picked out for subsequent investigations due to its higher knockdown efficiency. Transwell migration and wound healing assays revealed that hypoxia markedly facilitated cell migration, while IGF2BP3 knockdown suppressed the increase of cell migratory ability induced by hypoxia in MKN-45 and HGC-27 cells (**Figures 2C, D**). It has been well documented that hypoxia-induced tumor metastasis is closely correlated with angiogenesis (8). Moreover, Yang et al. demonstrated that IGF2BP3 could positively regulate angiogenesis in colon cancer (31). Thus, the effect of IGF2BP3 depletion on VEGF (a key angiogenic factor) secretion was examined in SC cells exposed to hypoxia. Results showed that IGF2BP3 depletion inhibited hypoxia-induced VEGF secretion in MKN-45 and HGC-27 cells (**Figure 2E**). Next, the alteration of angiogenic ability was further assessed by HUVEC tube formation assay *in vitro* (32). As presented in **Figure 2F**, there was a noticeable elevation of HUVEC tube formation capacity in the hypoxia-treated group relative to the normoxia-treated group. Furthermore, reduced tube formation ability was noticed in HUVECs cultured in the conditioned medium from MKN-45 or HGC-27 cells with IGF2BP3 loss and hypoxia treatment compared to those maintained in the conditioned medium from corresponding hypoxia-treated SC cells without IGF2BP3 depletion (**Figure 2F**).

### HIF1A as a Potential IGF2BP3 Target Was Highly Expressed in SC

**Figure 3:**
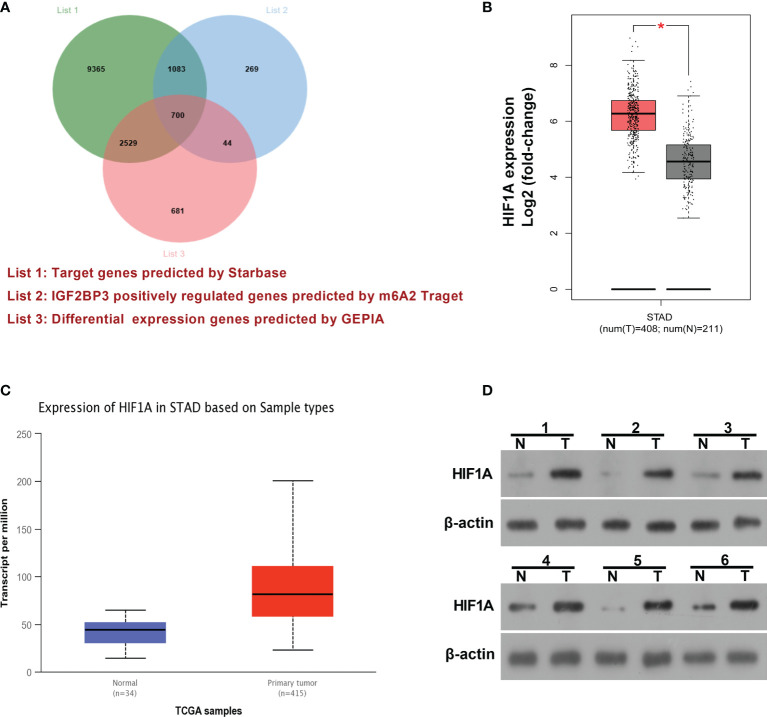
HIF1A as a potential IGF2BP3 target was highly expressed in SC. **(A)** Venn analysis (http://jvenn.toulouse.inra.fr/app/example.html) for genes in **Supplementary Tables 1**, **2**, and **3**. List 1: Starbase-predicted mRNAs that could bind with IGF2BP3. List 2: m6A2Target-analyzed genes that could be positively regulated by IGF2BP3. List 3: GEPIA-analyzed differentially expressed genes in SC tissues *versus* normal tissues. **(B)** Expression analysis of HIF1A in 408 cases of SC tumor samples and 211 cases of normal samples by the GEPIA website (http://gepia.cancer-pku.cn/). **(C)** Expression analysis of HIF1A in primary SC tumor tissues (n=415) and normal tissues (n=34) by the UALCAN database (http://ualcan.path.uab.edu/). **(D)** Expression level of HIF1A in 6 pairs of SC tumor tissues and adjacent normal tissues was determined by western blot assay. ^*^ *p* < 0.05.

IGF2BP3 has been identified as an RNA binding protein over the past decades. Hence, mRNAs that could bind with IGF2BP3 were predicted by the Starbase database (http://starbase.sysu.edu.cn/) and presented in **Supplementary Table 1** (List 1). Moreover, genes that could be positively regulated by IGF2BP3 were downloaded from m6A2Target (http://m6a2target.canceromics.org) and shown in **Supplementary Table 2** (List 2). To further screen out potential IGF2BP3 targets implicated in the pathogenesis of SC, differentially expressed genes (|log_2_FoldChange| > 1) in SC tissues *versus* normal samples were downloaded from the GEPIA website (http://gepia.cancer-pku.cn/) and displayed in **Supplementary Table 3** (List 3). Venn analysis for genes in **Supplementary Tables 1**, **2**, and **3** revealed that 700 differentially expressed genes in SC had the probability to be directly and positively regulated by IGF2BP3 (**Figure 3A** and **Supplementary Table 4**). Among these 700 genes, HIF1A was selected for further explorations because it has been well documented to be a crucial factor in hypoxic responses and angiogenesis (33, 34). GEPIA analysis showed that HIF1A expression level was noticeably increased in SC tumor tissues (red, T) than that in normal tissues (gray, N) (**Figure 3B**). Moreover, the expression pattern of HIF1A in SC was further analyzed by the UALCAN database (http://ualcan.path.uab.edu/). UALCAN analysis revealed that HIF1A was highly expressed in primary SC tumor tissues relative to normal tissues (**Figure 3C**). Moreover, western blot assay further demonstrated that HIF1A expression level was markedly elevated in SC tissues (n=6) than that in corresponding adjacent normal tissues (n=6) (**Figure 3D**).

### IGF2BP3 Positively Regulated HIF1A Expression in an m6A-Dependent Manner

**Figure 4:**
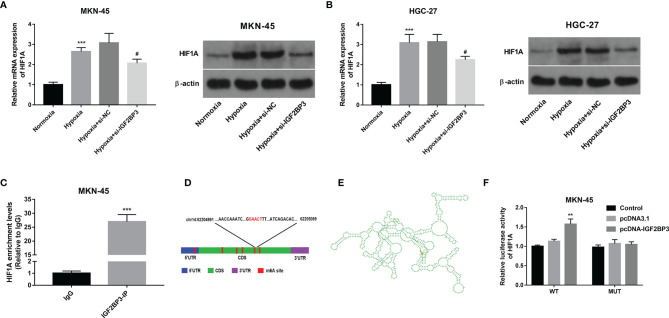
IGF2BP3 positively regulated HIF1A expression in an m6A-dependent manner. **(A, B)** MKN-45 and HGC-27 cells were transfected with si-NC or si-IGF2BP3#1 for 48 h and then maintained in hypoxic conditions for another 24 h Cells in the normoxia group were maintained in normoxic conditions for 72 h Cells in the hypoxia group were cultured in normoxia for 48 h and then exposed to hypoxia for an additional 24 h Next, HIF1A mRNA and protein levels were measured through RT-qPCR and western blot assays, respectively. **(C)** RIP assay was performed using IgG or IGF2BP3 antibody in MKN-45 cells. HIF1A level in IgG or IGF2BP3 immunoprecipitation complex was measured through RT-qPCR assay. **(D, E)** Putative m6A modification and IGF2BP3 binding site on HIF1A mRNA. **(F)** Luciferase activities were measured at 48 h post-transfection. ^**^ *p* < 0.01, ^***^ *p* < 0.001, ^#^ *p* < 0.05.

Next, RT-qPCR and western blot assays demonstrated that hypoxia-induced HIF1A expression and IGF2BP3 knockdown inhibited the increase of HIF1A expression induced by hypoxia in MKN-45 and HGC-27 cells (**Figures 4A, B**). Subsequent RIP assay revealed that HIF1A could be substantially enriched by IGF2BP3 antibody in MKN-45 cells, suggesting that IGF2BP3 could bind with HIF1A mRNA (**Figure 4C**). Next, the m6A modification sites of HIF1A mRNA were predicted by the SRAMP website (https://www.cuilab.cn/sramp). Combined with the SRAMP-predicted HIF1A m6A modification sites and Starbase-predicted IGF2BP3 binding sites on HIF1A mRNA, we found that there was a high confidence HIF1A m6A site among Starbase-predicted IGF2BP3 binding sites. The position of this site within HIF1A mRNA was displayed in **Figure 4D** and the local structure of this site was visualized in **Figure 4E**. Based on the above-mentioned data, we supposed that this specific m6A site might play an essential role in mediating the m6A-dependent interaction of m6A reader IGF2BP3 and HIF1A mRNA. To further validate this speculation, a wild-type (WT) or mutant type (MUT) HIF1A luciferase reporter containing this putative or mutant site was constructed, respectively. Subsequent luciferase reporter assay demonstrated that IGF2BP3 overexpression led to the notable increase of luciferase activity of WT HIF1A reporter, but did not influence the luciferase activity of MUT HIF1A reporter in MKN-45 cells (**Figure 4F**). In summary, these outcomes showed that IGF2BP3 positively regulated HIF1A expression in an m6A-dependent manner.

### IGF2BP3 Exerted Its Functions by Up-Regulating HIF1A in SC

**Figure 5:**
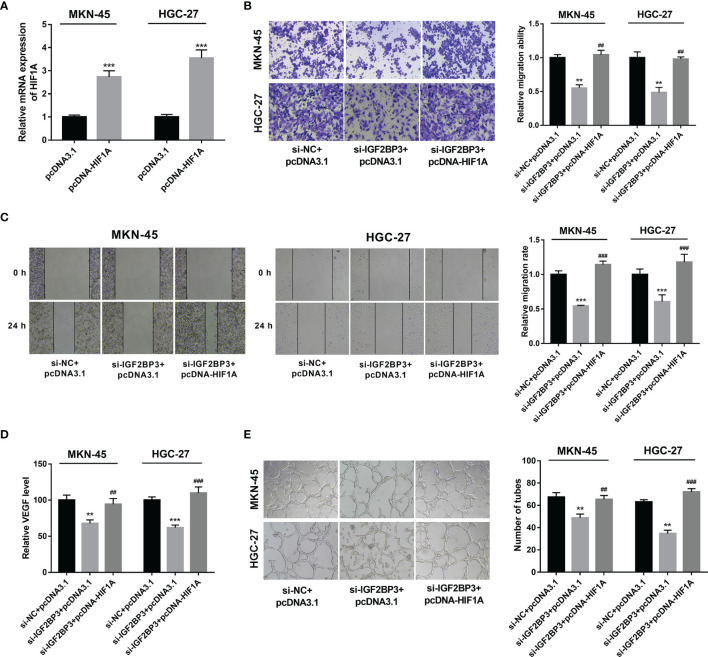
IGF2BP3 exerted its functions by up-regulating HIF1A. **(A)** MKN-45 and HGC-27 cells were transfected with pcDNA3.1 or pcDNA-HIF1A. Next, HIF1A mRNA level was measured by RT-qPCR assay at 48 h upon transfection. **(B–E)** MKN-45 and HGC-27 cells were transfected with si-NC+pcDNA3.1, si-IGF2BP3#1+pcDNA3.1, or si-IGF2BP3#1+pcDNA-HIF1A for 48 h and then maintained in hypoxic conditions for another 24 h, followed by the examination of cell migratory potential **(B, C)** and VEGF secretion level **(D)**. **(E)** MKN-45 and HGC-27 cells were transfected with si-NC+pcDNA3.1, si-IGF2BP3#1+pcDNA3.1, or si-IGF2BP3#1+pcDNA-HIF1A for 48 h and then maintained in hypoxic conditions for another 24 h, followed by the collection of conditioned medium. Next, HUVECs were cultured in a mixed medium of ECM and conditioned medium (volume ratio=1:1) and tube formation potential was examined at 12 h after incubation. ^**^ *p* < 0.01, ^***^ *p* < 0.001, ^##^ *p* < 0.01, ^###^ *p* < 0.001.

Next, RT-qPCR assay demonstrated that the transfection of pcDNA-HIF1A triggered the notable up-regulation of HIF1A expression level in MKN-45 and HGC-27 cells (**Figure 5A**). Moreover, HIF1A overexpression alleviated the detrimental effects of IGF2BP3 loss on cell migration and VEGF secretion in MKN-45 and HGC-27 cells exposed to hypoxia (**Figures 5B–D**). Additionally, there was a noticeable improvement of tube formation capacity in HUVECs cultured in the medium from MKN-45 and HGC-27 cells with IGF2BP3 depletion and HIF1A overexpression compared with those maintained in medium from matching cells with IGF2BP3 loss alone under hypoxic conditions (**Figure 5E**), suggesting that IGF2BP3 loss in hypoxia-exposed SC cells suppressed the tube formation of HUVECs by down-regulating HIF1A.

### IGF2BP3 Knockdown Inhibited Hypoxia-Induced Angiogenesis by Down-Regulating HIF1A *In Vivo*

**Figure 6:**
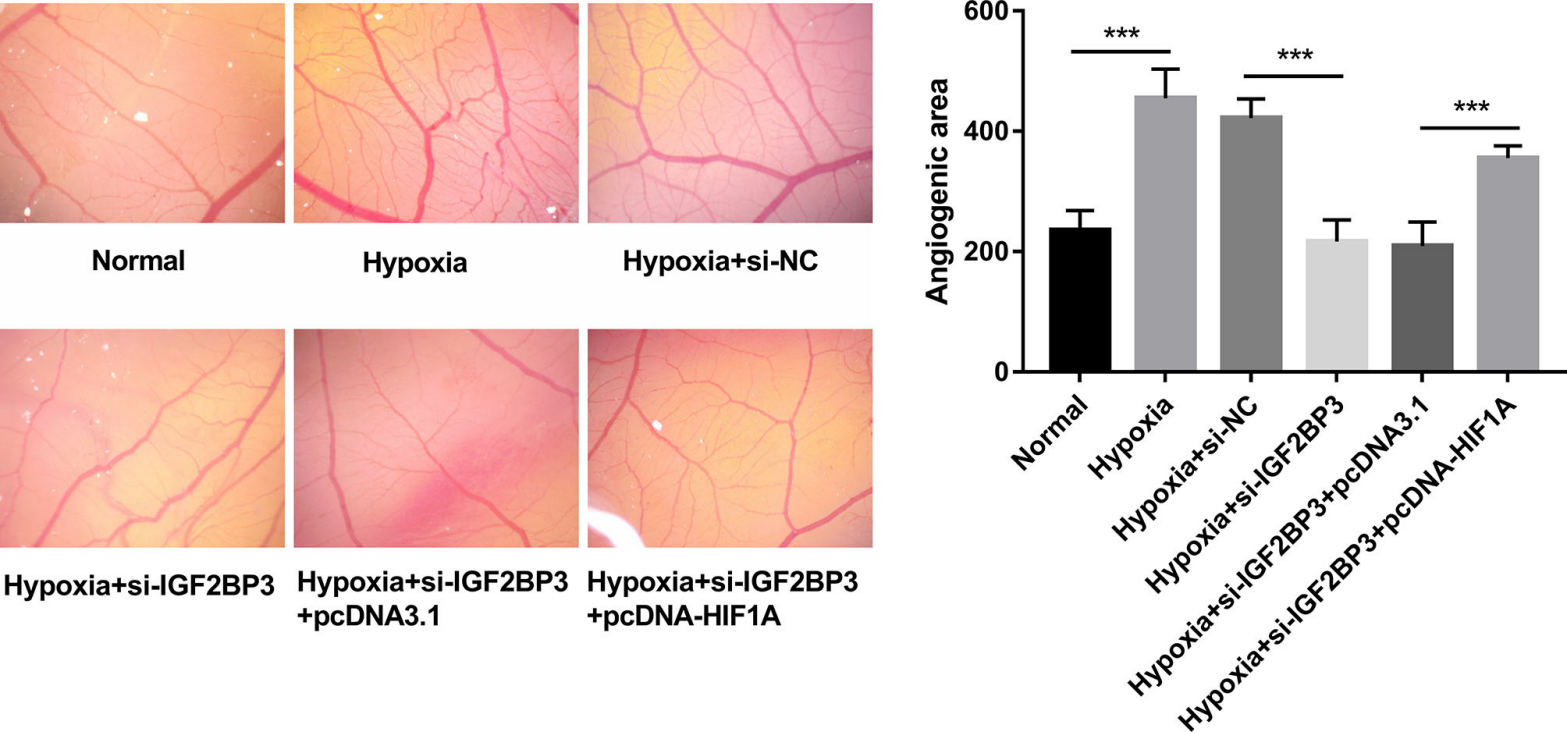
IGF2BP3 knockdown inhibited hypoxia-induced angiogenesis by down-regulating HIF1A *in vivo*. MKN-45 and HGC-27 cells were transfected with si-NC, si-IGF2BP3#1, si-IGF2BP3#1+pcDNA3.1, or si-IGF2BP3#1+pcDNA-HIF1A for 48 h and then maintained in hypoxic conditions for another 24 h. Cells in the normoxia group were maintained in normoxic conditions for 72 h. Cells in the hypoxia group were cultured in normoxia for 48 h and then exposed to hypoxia for an additional 24 h. Next, the conditioned medium was collected and added to the exposed CAM. The angiogenic ability was assessed at 3 days after incubation. ^***^ *p* < 0.001.

CAM assay has been widely used to investigate the alteration of angiogenic potential *in vivo* (35). In this text, the effect of the IGF2BP3/HIF1A axis on angiogenesis was further investigated by CAM assay *in vivo*. Results showed that IGF2BP3 loss hindered hypoxia-induced angiogenesis, and HIF1A overexpression weakened the inhibitory effect of IGF2BP3 loss on hypoxia-induced angiogenesis *in vivo* (**Figure 6**). That was to say, IGF2BP3 facilitated angiogenesis by up-regulating HIF1A in SC under hypoxic conditions.

## Discussion

Over the past decades, IGF2BP3 has been identified as a promising diagnostic/prognostic biomarker or therapeutic target in multiple cancers (21, 24, 36). However, it is still far from clinical application due to a host of unsolved molecular and technical issues (24). For example, the downstream effectors and regulatory mechanisms by which IGF2BP3 exerts its roles in cancer tumorigenesis and progression have not been well documented (24). Also, the knowledge of the interaction between IGF2BP3 and tumor hypoxic microenvironment is quite limited (24). Moreover, the m6A-dependent targets of IGF2BP3 remain largely unknown to date. A deep insight into the complex interactions between IGF2BP3 and tumor hypoxic microenvironment or its m6A-dependent targets might contribute to the clinical use of IGF2BP3.

In this project, our data revealed that m6A “reader” IGF2BP3 was highly expressed in SC. Consistent with our study, IGF2BP3 also has been reported to be highly expressed in SC tissues (25, 28). Moreover, we demonstrated that IGF2BP3 expression was noticeably increased in SC cells exposed to hypoxia. Functional analysis revealed that IGF2BP3 knockdown inhibited hypoxia-induced cell migration and angiogenesis in SC. These outcomes suggested IGF2BP3 might be partly responsible for the metastasis of SC under hypoxic conditions.

Recently, IGF2BP3 has been identified as an m6A “reader” that can control the fates of target mRNAs by recognizing the m6A motifs of RNAs (37). For instance, IGF2BP3 enhanced the proliferative activity of clear cell renal cell cancer cells by increasing the expression of cyclin-dependent kinase 4, collagen type VI alpha 1 chain, laminin subunit alpha 5, and fibronectin 1 in an m6A-dependent manner (38). IGF2BP3 facilitated transmembrane BAX inhibitor motif containing 6 (TMBIM6) expression by increasing the m6A level of TMBIM6 mRNA in laryngeal squamous cell cancer (39). Thus, potential m6A targets of IGF2BP3 in SC were searched based on the existing databases and websites. Among these possible targets, HIF1A was selected for further investigations given its central roles in the hypoxic tumor microenvironment (40).

Over the past decades, HIF1A has been well documented as a major hypoxia-responsive factor (40). In a hypoxic tumor microenvironment, HIF1A signaling is activated and activated HIF1A signaling can regulate the biological responses related to tumorigenesis and progression (*e.g.* tumor cell survival, proliferation, metastasis, and angiogenesis) in multiple cancers including SC (41, 42). Moreover, HIF1A expression was found to be associated with the poor prognosis of patients with SC (43, 44). Additionally, previous studies showed that HIF1A loss inhibited the increase of cell migratory and invasive capacities induced by hypoxia in SC (45, 46). HIF1A/VEGF signaling pathway also has been found to be involved in mediating SC tumor growth and angiogenesis under hypoxic conditions (47, 48).

In this text, we demonstrated that HIF1A was a target of IGF2BP3 and IGF2BP3 could positively regulate HIF1A expression in SC cells by directly binding to a specific m6A site in the CDS of HIF1A mRNA. Moreover, HIF1A overexpression alleviated the detrimental effects of IGF2BP3 loss on hypoxia-induced cell migration and angiogenesis in SC.

Taken together, our outcomes showed that IGF2BP3 knockdown inhibited hypoxia-induced cell migration and angiogenesis by down-regulating HIF1A in an m6A dependent manner in SC. This is the first study to elucidate the biological functions of the IGF2BP3/HIF1A axis in hypoxia-induced adaptive responses and hypoxia-triggered tumor malignant progression in SC. Moreover, our study demonstrated that IGF2BP3 could directly bind to an m6A site in the CDS of HIF1A mRNA in SC cells. Given the critical roles of hypoxia in intratumor heterogeneity, tumor aggressive progression, immune escape, and therapeutic resistance, an in-depth elaboration of hypoxia-related regulatory networks might contribute to the better management of the aforementioned troublesome problems in cancers.

## Data Availability Statement

The original contributions presented in the study are included in the article/**Supplementary Material**. Further inquiries can be directed to the corresponding author.

## Ethics Statement

The studies involving human participants were reviewed and approved by the Research Ethics Committee of the First Affiliated Hospital of Zhengzhou University. The patients/participants provided their written informed consent to participate in this study.

## Author Contributions

LJ designed and performed the experiments and wrote the manuscript. YL, YH, and DW contributed to experimental work and data analysis. LY conducted the experiments. HW revised the manuscript. All authors contributed to the article and approved the submitted version.

## Conflict of Interest

The authors declare that the research was conducted in the absence of any commercial or financial relationships that could be construed as a potential conflict of interest.

## Publisher’s Note

All claims expressed in this article are solely those of the authors and do not necessarily represent those of their affiliated organizations, or those of the publisher, the editors and the reviewers. Any product that may be evaluated in this article, or claim that may be made by its manufacturer, is not guaranteed or endorsed by the publisher.

## Supplementary Material

Supplementary Table 1 Starbase-predicted mRNAs that could bind with IGF2BP3.

Supplementary Table 2 m6A2Target-analyzed genes that could be positively regulated by IGF2BP3.

Supplementary Table 3 GEPIA-analyzed differentially expressed genes in SC tissues *versus* normal tissues.

Supplementary Table 4 Venn analysis for genes in **Supplementary Tables 1**, **2** and **3** revealed that 700 differentially expressed genes in SC had the probability to be directly and positively regulated by IGF2BP3.
